# Symptom experience and perceived symptom manageability among people with HIV in China: a phenomenological study

**DOI:** 10.3389/fpsyg.2025.1422509

**Published:** 2026-01-23

**Authors:** MeiLian Xie, AiPing Wang, ZhaoXia Lin, ZhiYun Zhang, KeRong Wang, YanPing Yu, XiaoJing Ma, ZhengLi Yu, JianXue Ke

**Affiliations:** 1 Nursing Management Department, Beijing Ditan Hospital, Capital Medical University, Beijing, China; 2 Nursing Management Department, Capital Center for Children's Health, Capital Institute of Pediatrics, Capital Medical University, Beijing, China; 3 School of Nursing, China Medical University, Shenyang, Liaoning, China; 4 Beijing Home of Red Ribbon, Beijing Ditan Hospital, Capital Medical University, Beijing, China; 5 Infection Center, Beijing Ditan Hospital, Capital Medical University, Beijing, China

**Keywords:** HIV, symptom experiences, symptom manageability, people with HIV, qualitative research

## Abstract

**Background:**

Symptoms play an important role in the evolution of the patient’s disease and their quality of life among people with HIV (PWH). This study aimed to explore the Journey of symptoms experienced and perceived symptom manageability (PSM) among PWH under the Chinese Cultural Context.

**Methods:**

A qualitative, phenomenological study was conducted at an HIV-designated medical institution in mainland China. From April 2021 to June 2021, in-depth interviews with 11 PWH and focus group interviews with 6 nurses working in HIV wards were conducted, and their related experiences, attitudes, and coping were studied and deeply described. Data collection and analysis were carried out simultaneously by two researchers, respectively.

**Results:**

Based on the research scope, two primary themes and five sub-themes were extracted and refined from the perspectives of the HIV/AIDS population and clinical nursing staff. Each theme encompasses several sub-themes: Theme 1: Perception of Symptom Manifestation includes the following sub-themes: Sub-theme 1-1: Physical and Perceptual Dimensions of Symptom Experience (Sub-theme 1-1-1: Distress Rooted in Recurrent and Persistent Symptoms; Sub-theme 1-1-2: Confusion Regarding the Different Origins of the Same Symptoms); Sub-theme 1-2: Anticipatory Anxiety About Symptom Trajectories (Sub-theme 1-2-1: Anxiety Regarding Symptom Progression and Dynamic Evolution; Sub-theme 1-2-2: Collapse Following the Concurrent Interaction of Multiple Symptoms); Sub-theme 1-3: Adaptive Tension in Symptom Management (Sub-theme 1-3-1: Comparison Between Self-Perception and Clinical Objective Indicators; Sub-theme 1-3-2: The Struggle Between Exhaustion and the Will to Survive). Theme 2: Perception of Symptom Manageability—Harmonious Unity of Heaven, Earth, and Humanity consists of the following sub-themes: Sub-theme 2-1: Perception of Self-Worth; Sub-theme 2-2: Perception of the Value of Social Support Systems (Sub-theme 2-2-1: Self-Awareness and Self-Regulation; Sub-theme 2-2-2: Self-Coping with Symptoms; Sub-theme 2-2-3: Growth Under the Care of the State and Society; Sub-theme 2-2-4: Hope Evoked by Medical Care and Nursing; Sub-theme 2-2-5: The Contradictory Nature of Resource Supply and Demand); Sub-theme 2-3: Perception of the Value of Interpersonal Interaction Systems (Sub-theme 2-3-1: The Motivation of Love and Being Loved; Sub-theme 2-3-2: Satisfaction After Reflection on Conflicting Events; Sub-theme 2-3-3: Exemplification of Successful Role Models; Sub-theme 2-3-4: The Burden Within the Culture of Illness).

**Conclusion:**

The manifestation of symptoms, coping strategies, and depictions of the concept of PSM among PWH are intricately intertwined with individual characteristics, life experiences, cultural backgrounds, and other factors. This study provides a systematic exposition and conceptual delineation of PSM. By elucidating culture-specific coping strategies and their implications for HIV care, this research significantly advances the understanding of PSM within the Chinese context. This lays the groundwork for an exploratory study into the intrinsic motivational mechanisms underlying the perception of symptom manageability in the future.

## 1 Introduction

HIV has still been a major public health problem threatening human health worldwide (Yuan and Han, 2019). Although international progress on HIV research and treatment improves the survival rate, PWH may still experience multiple symptoms caused by HIV infection, the short or long-term side effects of HIV medications, and comorbidities (Kuan et al., 2023). These physical and psychological symptoms seem not to be relieved with medical care existing (Bratton et al., 2017; Izydorczyk et al., 2019; Schreiner et al., 2020). Perception of symptoms may outweigh cold laboratory numbers because of the social nature of human beings and systematic development of body and mind (Ruiz-Burga et al., 2023). Studies have shown that symptoms play an important role in the evolution of the patient’s disease and their quality of life among people with HIV (PWH) (Polanka et al., 2021; Pujasari et al., 2021). Additionally, the escalating cycle of symptoms heightens its noxious, bothersome, and distressing quality, increasing in symptom intensity; more numerous symptoms; symptoms that are now more bothersome, distressing, and intrusive; and the strengthened conviction that one is sick (Barsky and Silbersweig, 2022).

During the past decades, several studies demonstrated that most PWH experience multiple symptoms, which are likely to have a negative impact on their quality of life, functional performance, and adherence or burden to treatment among PWH (Zheng et al., 2021; Zhu et al., 2019). Xie investigated 493 PWH in China and found that those with symptoms have a worse health-related QOL compared to asymptomatic individuals and the general public (Xie et al., 2023). Olson et al. also revealed that physical symptoms, particularly those related to appearance and sexual functioning, most strongly predicted HRQoL (Olson et al., 2019). Another study demonstrated that high levels of symptom severity are associated with higher levels of cumulative treatment burden and task-specific (medication and physical activity) burden in PWH (Schreiner et al., 2020). In China, Zhu and colleagues identified symptom clusters and explored symptom networks among PWH (Hongli et al., 2021; Zhu et al., 2021a; Zhu et al., 2021b).

While the quantity and severity of symptoms may be captured using numbers, symptoms can also imbue individuals with different perceptions, understandings, and attitudes within specific cultural contexts. It is these nuances that require individuals to voice their experiences to generate clinically actionable insights. Therefore, grounding symptom assessment in established theoretical frameworks is critical for effective management. Here, we operationalize Perceived Symptom Manageability (PSM) through the lens of the Self-Regulatory HIV Symptom Management Model (SSMM-HIV) (Fierz et al., 2013) as the extent of the perceived ability to bring social and personal resources into play to successfully deal with/control symptoms, despite difficulties. This framework positions PSM as a pivotal mediator between self-regulation efforts and health outcomes (Xie et al., 2024). Specifically, Xie et al. validated SSMM-HIV’s proposition that PSM mediates the pathway from symptom perception to quality of life among Chinese PWH. Symptom manageability was introduced as a variable representing the cognitive and emotional evaluation of individual symptom management endeavors, addressing the overall success of these actions as perceived by the patient. This perception will, in turn, affect subsequent actions, health-related quality of life, clinical parameters, and adherence. Despite SSMM-HIV’s theoretical emphasis, the subjective construction of manageability—particularly how patients conceptualize resource mobilization barriers or success—remains unexplored in naturalistic contexts. Qualitative research could provide in-depth insights and understanding of real-world problems in contrast to quantitative research (Denny and Weckesser, 2019). Understanding what patients think, feel, or do in their natural context can make clinical practice and evidence-based interventions more effective, efficient, equitable, and humane (Korstjens and Moser, 2017). However, previous qualitative studies only described symptom attributions, symptom influence on life or others, and coping with adverse symptoms (Iribarren et al., 2018; Mankhokwe et al., 2023; Nyongesa et al., 2022). For example, Iribarren identified 8 self-management strategies to address symptoms. Mankhokwe’s study revealed that these symptoms hampered participants’ ability to walk, work, conduct business, perform household chores, and care for children. Previous studies, often conducted in resource-rich settings or focusing broadly on experience rather than manageability, have not deeply explored this specific construct using rigorous phenomenological approaches. However, under the influence of China’s 5000-year medical culture, there has been limited in-depth exploration of the physical and psychological trauma experienced by PWH when confronted with various symptoms, as well as the coping strategies they employ. Our study directly addresses these gaps by using a phenomenological lens to elucidate the lived experience of symptom manageability within the unique socio-cultural context of contemporary China, thereby helping medical professionals understand the trajectory of symptoms from the patient’s perspective and clarify the daily interpretation of symptom manageability. These questions necessitate a phenomenological design, which is optimal for capturing the pre-reflective, embodied dimensions of ‘manageability’—a concept inherently tied to subjective sense-making processes that remain unexplored in prior mechanistic accounts of symptom management. Our study, adopting a phenomenological perspective, directly addresses existing research gaps. It illuminates the lived experiences of symptom manageability within the unique social and cultural context of contemporary China. This approach enables healthcare workers to understand symptom trajectories from the patients’ perspectives, clarifying the practical implications of symptom manageability in daily life, and enhancing the theoretical framework of SSMM-HIV within the Chinese cultural context. Consequently, it provides novel insights for future clinical practices in symptom care.

## 2 Methods

### 2.1 Study design and study settings

In this study, attention was given to exploring the perceptions, inner experiences, and individual understandings of the co-occurring symptoms among PWH, with emphasis on the subjective experiences and feelings regarding symptoms acquired by this population during the long-term coexistence of illnesses. Phenomenology is used to explore how individuals make sense of the world, providing insightful accounts of their subjective experiences (Korstjens and Moser, 2017), which was considered the most appropriate method for our study. Therefore, phenomenology was used to gain insight into the experiences or perceptions of PWH who have various symptoms, and healthcare professionals working in HIV/AIDS wards could depict their perception of symptom manageability in this study. If the interview from a patient perspective is a micro-level description, the healthcare provider’s views and understanding of symptom manageability can help us explain it from the macro level. A sample of 11 PWH consulted in ‘Beijing Home of Red Ribbon’ or hospitalized in the ward of ‘Beijing Ditan Hospital’ in China were enrolled in the study. A focus group interview was also conducted with nurses working in the HIV ward. This Hospital is one of the major medical institutions for HIV treatment in China, which is authorized as both the National Medical Center for Infectious Diseases and the WHO Centre for Integrated Management of AIDS Treatment and Care. Interviews were conducted between April 2021 and June 2021.

### 2.2 Study sample

For qualitative research, participants are selected based on their firsthand experience with a particular culture, social interaction, or interesting phenomenon. Qualitative research typically involves selecting research subjects and determining sample size based on the nature of the question, the requirements of relevant theories, and the richness of available data. The ability of interviewees to provide necessary information serves as a sampling criterion (Moser and Korstjens, 2022). Therefore, we identified cases that could articulate their experiences clearly and engage in self-reflection. In this regard, purposive sampling is more suitable for qualitative research, with an overarching principle of maintaining maximum flexibility and complementing homogeneity and maximum variation strategies. When selecting participants, demographic factors and disease-related variables should be considered. Additionally, to gain a multifaceted understanding of the issue and its practical significance, the study focused on conducting focus group interviews with nurses who care for PWH in medical institutions. Nurses were selected based on their experience in HIV care and variation in age, position or work duration. Declining participants uniformly declined demographic disclosure to protect privacy. This study determined the sample size based on the principles of data saturation in the analysis process and the information efficiency model in qualitative interviews (Hongling et al., 2021). Data saturation refers to the point at which new data collection and iterative analysis no longer yield novel insights or dimensions relevant to the research questions. Ultimately, 11 PWH and 6 nurses were interviewed.

#### 2.2.1 Semi-structured interviews with PWH

Eleven PWH were enrolled in the study. To be eligible for participation in the study individuals had to: (1) be diagnosed as HIV-infected; (2) be over 18 years old; (3) receive ART; (4) over 1 year after HIV diagnosis; (5) experience related symptoms during the past year;(6) informed consent. Exclusion criteria include (1) severe mental and neurological diseases; (2) those with other serious illnesses or in a life-threatening condition who are unable to participate in interviews (e.g., active malignancy, end-stage renal disease, decompensated liver cirrhosis, or severe cardiovascular disease); (3) those who cannot usually comprehend or respond to questions.

#### 2.2.2 Focus group with nurses

Six nurses were purposefully sampled and generated an informative focus group discussion with variation in age, and position status. Inclusion criteria: (1) At least 3 years’ work experience in the field of HIV; (2) working in the related wards or clinics in the last year; (3) informed consent. Elimination criteria: those who withdraw from the interview due to various reasons in the middle.

### 2.3 Recruitment

To ensure the representativeness and comprehensiveness of the participants, various recruitment strategies were employed in this section. These project team members served as the main recruiters of participants in this study. These recruiters included medical staff who had been actively involved in frontline HIV work in outpatient clinics and wards for an extended period. These members have established deep friendships and connections with PWH through their long-term interactions. Hence, their involvement ensures the efficiency of patient recruitment during the research process, eliminating barriers for the primary researchers or interviewers to enter the research context. They undergo uniform training and learning provided by the project team and, during their work in outpatient clinics or hospital wards, select eligible interviewees based on the recruitment criteria. Recruiters arranged the time and location for face-to-face interviews with the PWH and then informed the primary interviewers. Additionally, the primary interviewers were responsible for contacting the nurse managers in outpatient clinics and wards who were key interviewees in this study, explaining the need for the focus group interview, obtaining consent, and negotiating the time and place.

### 2.4 Data collection

#### 2.4.1 Semi-structured interviews

Data collection happened in the Red Ribbon Office(clinic) or HIV-related wards in ‘Beijing Ditan Hospital’ in China. The interviewer (MLX, ZXL) arrived at the designated interview location at the scheduled time. Regardless of whether the interview was in the clinic or the ward, the face-to-face interview was conducted in a quiet room with a ‘Do Not Disturb’ sign on the door. Following consent and assent, an audio-recorded interview lasting between 40 min and 1 h was conducted. After being recruited or during interviews, participants were informed of the interviewer’s specific situation and personal identity. The Principal Investigator (PI) (MLX), who was familiar with HIV-related fields but had not worked in these departments, and a research assistant (ZXL), who had worked as a nurse in HIV wards, conducted the interviews. During each interview, the PI was in charge of communicating with a participant based on an interview outline prepared in advance, as well as taking down ideas on site, and a research assistant observed and wrote some non-verbal characteristics of the participant, such as physical appearance, demeanor, and speech tone, to create a participant profile. All participants finished their interviews, although one sobbed midway through; they still showed a desire to continue the interview after being properly pacified. Participants were asked to discuss not only the impacts and experiences of symptoms, but also their attitudes, beliefs, and behaviors related to coping and managing symptoms. Questions were asked about the most distressing symptom, self-regulation or self-management, support from outside, experience of coping successfully, and so on.

#### 2.4.2 Focus group with healthcare professionals

A focus group of nurses was invited to participate in a discussion to complement understanding of patient-related issues. Due to time constraints, we conducted this interview in the nurses’ lounge at noon. After informed consent, an audio recording and field notes were taken, which lasted almost 1 h. The two interviewers (MLX, ZXL) conducted a focus group interview in the same way as the patients’ data collection process. Nurses were asked about common problems with patients, such as whether they talked about symptoms with you. How well are they coping with their symptoms, and how are their attitudes toward them? And the role of nursing in symptom management among PWH?

### 2.5 Transcription and coding

Reflexivity was maintained through weekly team debriefings documenting how professional backgrounds influenced interpretation. Clinical researchers recorded presuppositions in reflexive journals, which were systematically challenged during analysis. All coding decisions were reviewed by external qualitative methodologists unaffiliated with the clinical sites. Data collection and analysis were conducted simultaneously by two well-trained researchers (MLX, ZXL). Audio recordings of interviews were transcribed verbatim and anonymized (Serial numbers were used) within 24 h after the end of each interview, and the contents of the tapes were converted into Word documents. Then, the follow-up analysis of the data will be completed within 72 h after each interview, and the analysis process will be carried out by two researchers (MLX, ZXL) using manual methods. When doubts arose, a third researcher (APW) was invited to evaluate the findings until a unified and consistent thematic framework was finally established. Transcripts were analyzed using the Colaizzi’s seven-step data analysis method (Sanders, 2003): (1) The researcher meticulously recorded and carefully reviewed the interview data from the 11 participants and nurses; (2) Relevant and meaningful statements corresponding to the symptom experiences and manageability of PWH were extracted; (3) 8 ~ 13 key statements were then summarized and refined from every transcript; (4) Common characteristics or concepts among the meaningful statements were identified, forming two groups of themes and nine structured sub-themes; (5) These themes were closely linked to the research phenomenon and described in detail through researcher’ meeting; (6) All results were integrated to describe symptom experiences and manageability and provide a detailed account of the essential structure constituting this phenomenon; (7) We let all participants provide their contact to make sure that the results were returned to them via email or mail to verify the authenticity of the content. After conducting the preliminary analysis of the data and before final framework development, we contacted interviewees using the contact information provided. We invited them to review the transcription and themes to further supplement and correct the report. Data collection and analysis were conducted simultaneously, and data saturation was achieved when no new topics emerged. Study validity was enhanced through the use of deviant-case analysis and reflection on its impact on the process. Intercoder reliability was quantified via Cohen’s *κ* (pre-consensus *κ* = 0.78). Discrepancies underwent blind re-review, consensus meetings, and third-researcher arbitration until full agreement (*κ* = 1.00) was achieved. An audit trail documents all analytical decisions.

### 2.6 Ethics

Ethical approval was obtained from the Institutional Review Board of ‘Beijing Ditan Hospital’, with reference number ‘DTZZLX-202106’, where the study was conducted. We obtained written informed consent from all participants. To ensure data confidentiality, all interviews were conducted in private rooms at HIV-designated clinics or wards. Identifiers (names, locations, workplaces) were replaced with generic descriptors, and voice distortion software was applied to audio files before external transcription. All raw data and paper materials (consent forms, notes) were kept in double-locked cabinets accessible only to the PI. The participants’ informed consent was stored and managed separately from other personal information. At the conclusion of the interview, each participant was given a daily care kit as compensation for their participation in the study. We also provided psychological support at any time to ease participants’ anxiety during these interviews.

## 3 Results

A total of 11 individual semi-structured interviews and one focus group interview were conducted. Each session lasted from over 4 min to approximately 1 h. The coding process was carried out independently and back-to-back, followed by cross-review. Eleven patients were interviewed, and all were admitted to ‘Beijing Ditan Hospital’. Among them, five were from the Clinic, and the other six were from the Infectious Diseases Ward. These participants ranged in age from 27 to 63 years, with the average age being (45.00 ± 12.28) years. Six nurses participating in the focus group are all female, RN (Registered Nurses) from the People’s Republic of China, and have worked in HIV wards for at least 3 years. Additionally, detailed demographic information is presented in Tables 1–3.

**Table 1:** Participant characteristics (*N* = 11).

Characteristics	Frequency(n)/X ± S
Age	45.00 ± 12.275
Gender	
Male	8
Female	3
Race/ethnicity	
Han	9
Minority	2
Education level	
Middle school or below	1
High school or equivalent	5
University degree or equivalent	4
Master’s or above	1
Marital status	
Single	3
Married or Cohabitation	5
Divorced	0
Separated	1
Widowed	1
Employment status	
Student	1
On the job	7
Unemployment	1
Freelancer	0
Retirement	2
The number of permanent residents in the household	
One	2
Two	2
Three	6
Four or above	1
Living situation	
Alone	3
With family	5
Or with friendsor classmates	2
Otherwise	1
Region	
Urban	8
Rural	3
Years of HIV diagnosis (years)	
1 ~ 5	1
6 ~ 10	2
>10	8
Confirmed ways	
Hospital examination	6
Routine check-up	3
Self-inspection	1
Premarital check-up	0
Otherwise	1
ART use	
Yes	11
Years of ART use (years)	
1 ~ 5	1
6 ~ 10	2
>10	8
Disease staging	
Acute infection period	0
Asymptomatic	4
AIDS	7
CD4^+^ T cell count	
<200	2
200 ~ 499	2
>500	7
Having comorbidities	
Yes	9
No	2

**Table 2:** Participant information: PWH.

Number	Age	Sex	Race	Education level	Marital status	Employment status	Disease stage	CD4 + T count	Complication
P01	30	Male	Tujia	Undergraduate	Single	On the job	AIDS stage	<200	Pulmonary tuberculosis
P02	44	Male	Han	Undergraduate	Married	On the job	Asymptomatic	≥500	Hypertension
P03	32	Male	Han	High school	Single	On the job	AIDS stage	≥500	No
P04	63	Male	Han	Undergraduate	Separated	Retirement	AIDS stage	200 ~ 499	Coronary artery disease
P05	40	Male	Manchu	High school	Single	On the job	Asymptomatic	≥500	No
P06	49	Female	Han	High school	Married	Retirement	AIDS stage	≥500	Optic nerve injury
P07	57	Female	Han	High school	Married	Unemployment	AIDS stage	≥500	Reflux esophagitis/superficial gastritis
P08	57	Female	Han	Middle school	widowed	On the job	Asymptomatic	≥500	Hyperlipidemia
P09	27	Male	Han	Middle school	Single	Students	AIDS stage	≥500	Old cerebral infarction
P10	34	Male	Han	Undergraduate	Single	On the job	AIDS stage	≥500	Fatty liver
P11	49	Male	Han	High school	Single	On the job	AIDS stage	<200	Tuberculosis/pulmonary infection/syphilis

**Table 3:** Participant characteristics: healthcare professionals (*N* = 6).

Characteristics	Frequency(n)/X ± S
Age	28.83 ± 3.764
Working number of year	7.17 ± 4.355
Gender	
Male	0
Female	6
Education level	
High school or equivalent	1
University degree or equivalent	5

This study adopted a blended perspective that incorporates both patient and clinical nursing staff perspectives. Based on the research framework, 9 themes were distilled, focusing on perceptions of symptom manifestation and symptom manageability. Each theme is further divided into several subthemes, with the frequency of occurrence among respondents detailed in Table 4. Patients are identified by the label “P,” while clinical nursing staff are determined by the label “N,” followed by their respective reference numbers.

**Table 4:** Frequency of participant responses corresponding to sub-themes.

Themes, sub-themes		Exemplar quotes	Cases concordant with sub-theme content	Cases where sub-theme content is not mentioned in interview
Theme 1	Perception of symptom manifestation			
Sub-theme 1–1	Physical and perceptual dimensions of symptom experience			
Sub-theme 1-1-1	Distress rooted in recurrent and persistent symptoms	*When took related medicine at the beginning, I vomited a dozen times a day out of my control and still vomited no matter what I ate and drank. I vomited every day over a month.” (P03) “At the beginning, I always felt uncomfortable and wanted to defecate after dinner. It was intermittent all the time. I felt a little better only when eating nothing.*	11	0
Sub-theme 1-1-2	Confusing about the different origins of same symptoms	*Along with the kind of dizziness, I felt mixed up and very tired. Although enough rest, I was still fatigued. Everything would be linked to HIV. Does a disease cause an itch in my ears? Once diarrhea occurs, whether the disease itself is worsened or not?*	8	3
Sub-theme 1–2	Anticipatory anxiety about symptom trajectories			
Sub-theme 1-2-1	Anxiety regarding symptom progression and dynamic evolution.	*Sometimes on the arms, sometimes on the body, there were a lot of small red spots, which felt slightly itchy, that subsided within two or three days without medication. At the beginning, I did not care about it. After a period of time, I did not know why it eventually turned into eczema.*	8	3
Sub-theme 1-2-2	Collapse following the concurrent interaction of multiple symptoms	*I always have a fever and sweat at night with a lot of dreams, an exhausted feeling, and fading of physical strength, especially no vitality.” (P01)“I often have a stomachache and feel fatigue, even a fever for nearly a month or diarrhea over a week. Besides, my blood sugar increased inexplicably, and I had a splitting headache with rash, nausea, and vomiting. I used to be very thin, but now I am more than 200 kilograms, which makes me collapse.*	7	4
Sub-theme 1–3	Adaptive tension in symptom management			
Sub-theme 1-3-1	Comparison between self-perception and clinical objective indicators	*I pay more attention to my feelings. Sometimes the indicators are all good, but even if I feel uncomfortable, medical workers cannot find the reason, no matter how they check. However, the uncomfortable feeling is always there, which makes me distressed.*	9	2
Sub-theme 1-3-2	The struggle between exhaustion and the will to survive	*I try to control my psychological emotions and not link every situation to HIV/AIDS, but it’s indeed difficult. After all, we have to live with this disease for a long time. Once symptoms appear, we can still choose to seek medical treatment at the hospital. There’s always hope for a turnaround, which is better than having no solution at all.*	8	3
Theme 2	Perception of symptom manageability—harmonious unity of heaven, earth, and humanity.			
Sub-theme 2-1	Perception of self-worth			
Sub-theme 2-1-1	Self-awareness and self-regulation	*Without the support of my relatives and friends, I feel like I would have collapsed. And it touched me. I mean, it touched me. During the most difficult time, my family should be my biggest support, right? I can overcome any difficulties or barriers from the disease and gain great confidence in handling symptoms, owing to their support.*	10 + 6	1
Sub-theme 2-1-2	Self-coping with symptoms	*Getting enough sleep is good for alleviating discomfort and fatigue. If I am really tired, I would take a break on-site. For example, I might take a nap on a bus or in a taxi. I need to find something to distract myself, and I cannot think about it all the time. At that time, I remembered that I might just listen to the music constantly, and then inject my own emotions into the scene described by the music.*	11	0
Sub-theme 2-2	*Perception of the value of social support systems*			
Sub-theme 2-2-1	Growth under the care of the state and society	*I think a team that can organize some activities or lectures regularly, including sharing with each other about personal experiences, distress, and how to deal with symptoms, is very useful. Patients can learn a great deal from one another. I also took part in such activities, even though I worked as a volunteer, I think these groups are quite meaningful.*	11	0
Sub-theme 2-2-2	Hope evoked by medical care and nursing	*I am still looking forward to the development of science and technology, which can restore my health. At present, there are some better drugs explored, which bring fewer side effects than the previous ones.*	10 + 5	1
Sub-theme 2-2-3	The contradictory nature of resource supply and demand.	*Although I could choose to go to the hospital when I felt uncomfortable, it was a little troublesome and not very convenient. For example, if I had a slight symptom that puzzled me, a telemedicine service would be a better option than traveling to a distant location to see a doctor. I would leave a message for the doctor and discuss something with them. I think it’s convenient, and I also would not have to run so far.*	9	2
Sub-theme 2-3	Perception in social support			
Sub-theme 2-3-1	The motivation of love and being loved	*Without the support of my relatives and friends, I feel like I would have collapsed. And it touched me. I mean, it touched me. During the most difficult time, my family should be my biggest support, right? I can overcome any difficulties or barriers from the disease and gain great confidence in handling symptoms, owing to their support.*	11	0
Sub-theme 2-3-2	Satisfaction after reflection on conflicting events	*Looking! The condition of that patient in the ward next to mine is more serious than mine. I think I am lucky and have a good attitude. I will actively coordinate with the treatment and deal with any discomfort as soon as possible.*	7	4
Sub-theme 2-3-3	Exemplification of successful role models	*I seek out individuals in life who are particularly positive or have shown remarkable growth through their struggles. I draw inspiration from the development trajectories of these role models to motivate myself to move forward and persevere.*	6	5
Sub-theme 2-3-4	The burden within the culture of illness	*I needed to see a doctor when I felt uncomfortable and could not handle it by myself, but I dared not go to another hospital, because I could not tell others that I had AIDS.*	11	0

### 3.1 Theme 1: perception of symptom manifestation

PWH expressed varying levels of perception regarding their experiences of symptom occurrence, whether it’s the psychological anguish or the physical exhaustion they endure. Their vivid descriptions immerse us in the real world of this population, helping us gain insight, understanding, and empathy for the chaotic, conflicting, bewildering, and struggling process they went through.

#### 3.1.1 Sub-theme 1

Physical and perceptual dimensions of symptom experience.

##### 3.1.1.1 Sub-theme 1–1: distress rooted in recurrent and persistent symptoms

Symptoms in PWH often exhibit a pattern of recurrence and persistence. They find themselves trapped in an endless cycle of occurrence, relief, and relapse or enduring torment in prolonged states. Symptoms persist like a shadow, frequently visiting and severely impacting their physical and mental health. While many symptoms may not be life-threatening, they are challenging to control, exacerbating the sense of powerlessness and further catalyzing inner anguish. Torment and pain become the most frequent themes in the discourse of PWH. Behind this suffering lie countless lives compromised and helpless in their struggle against symptoms.

For example, “*When took related medicine at the beginning, I vomited a dozen times a day out of my control and still vomited no matter what I ate and drank. I vomited every day over a month.*” (P03) “*At the beginning, I always felt uncomfortable and wanted to defecate after dinner. It was intermittent all the time. I felt a little better only when eating nothing.*” (P04).

##### 3.1.1.2 Sub-theme 1–2: confusing about the different origins of the same symptoms

People with HIV describe a recurring phenomenon in their prolonged illness journey where the same symptom often occurs multiple times. Through continuous self-reflection and introspection, they realize that even though the same symptom recurs, the experience each time is not entirely identical. Therefore, they believe that the same underlying cause may not account for each instance of symptom occurrence. They often question themselves: Does HIV itself cause it? Could it be a side effect of medication? Or is it caused by other comorbidities? In any case, they are constantly troubled by this unknown confusion, seemingly unable to find its root cause. This anguish acts as an invisible shackle and restraint, layering and binding the PWH who are already trapped under the guise of HIV with further restrictions and constraints.

For example, “*Along with the kind of dizziness, I felt mixed up and very tired. Although enough rest, I was still fatigued. Everything would be linked to HIV. Does a disease cause an itch in my ears? Once diarrhea occurs, whether the disease itself is worsened or not?*” (P05).

#### 3.1.2 Sub-theme 2: anticipatory anxiety about symptom trajectories

##### 3.1.2.1 Sub-theme 2-1: anxiety regarding symptom progression and dynamic evolution

Symptoms are never static; through the accounts of the interviewees, we gain a deeper understanding that the symptoms affecting their physical and mental health also change over time. This dynamic development manifests in various ways: sometimes as an accumulation of symptoms, at other times as a reduction in the intervals between symptoms, and occasionally as an increase in the duration of symptoms or their severity. Along this trajectory of evolving changes, the interviewees consistently convey a sense of fear, panic, and anxiety, which intensifies as the symptoms progress and change. The interplay between the body and mind, coupled with the double impact, perpetuates a lingering and inescapable sense of anxiety.

For example, “*Sometimes on the arms, sometimes on the body, there were a lot of small red spots, which felt slightly itchy, that subsided within 2 or 3 days without medication. At the beginning, I did not care about it. After a period of time, I did not know why it eventually turned into eczema.*” (P04).

##### 3.1.2.2 Sub-theme 2-2: collapse following the concurrent interaction of multiple symptoms

The accounts of the interviewees present the current situation of concurrent multiple symptoms among PWH, revealing the catastrophic feeling they experience when multiple symptoms occur. They often feel on the verge of collapse and unable to bear the burden, as if teetering on the edge of life. The complex symptoms may be caused by HIV infection itself, comorbidities, or drug side effects. In any case, their symptoms are diverse, with physical discomfort intertwining with multiple issues such as mental, psychological, and emotional challenges. This interaction catalyzes the continuous exacerbation and deterioration of symptoms, leaving them unable to catch their breath and causing their lives to lose balance.

For example, “*I always have a fever and sweat at night with a lot of dreams, an exhausted feeling, and fading of physical strength, especially no vitality.” (P01) “I often have a stomachache and feel fatigue, even a fever for nearly a month or diarrhea over a week. Besides, my blood sugar increased inexplicably, and I had a splitting headache with rash, nausea, and vomiting. I used to be very thin, but now I am more than 200 kilograms, which makes me collapse.*” (P05).

#### 3.1.3 Sub-theme 3: adaptive tension in symptom management

##### 3.1.3.1 Sub-theme 3-1: comparison between self-perception and clinical objective indicators

In this interview, the interviewees expressed concern and emphasized the importance of self-perception. Throughout their lifespan, they often experience discomfort, even when related medical tests yield expected results. They still believe that experiencing discomfort indicates the presence of health issues that should be addressed through medical treatment or nursing intervention, rather than solely relying on laboratory test results or objective indicators. The interviewees’ self-perceived world contrasts with the clinical practice approach that traditionally relies more on objective measures, sparking a dialogue and contestation in the new era of HIV/AIDS management.

For example, “*I pay more attention to my feelings. Sometimes the indicators are all good, but even if I feel uncomfortable, medical workers cannot find the reason, no matter how they check. However, the uncomfortable feeling is always there, which makes me distressed.*” (P04).

##### 3.1.3.2 Sub-theme 3–2: the struggle between exhaustion and the will to survive

Most of the interviewees will talk about the toll that the long-term and recurrent onslaught of symptoms takes on their physical and mental well-being, as well as the depletion of their resources. However, at certain moments, they also reveal a determination to keep striving to survive, or the hope that comes from effective interventions. The tug-of-war between the hopelessness of the brink of life and the instinctual will to survive allows us to sense their inner struggles. Despite the exhaustion brought on by the onslaught of various symptoms, they exhibit a state of physical and mental fatigue. Yet, they strive to maintain a positive attitude for survival, persisting, and persisting again.

For example, *“I try to control my psychological emotions and not link every situation to HIV/AIDS, but it’s indeed difficult. After all, we have to live with this disease for a long time. Once symptoms appear, we can still choose to seek medical treatment at the hospital. There’s always hope for a turnaround, which is better than having no solution at all.”* (P05).

### 3.2 Theme 2: perception of symptom manageability—harmonious unity of heaven, earth, and humanity

In this study, as interviewees discussed their understanding of the issue, various dimensions and perspectives emerged, forming multiple themes categorized into perceptions of self-worth, social support systems, and interpersonal interaction systems. Additional insights from clinical nursing staff offered another perspective for examination and reflection. In this interview, the clinical nursing staff shared remarkably similar views with the interviewed patients regarding attitudes and perceptions of coping with symptoms, although with differing levels of significance. They believed that most PWH were gradually able to accept the reality of their illness and the frequent occurrence of various symptoms, enduring hardships, but ultimately became more accepting. Furthermore, clinical nursing staff also expressed the observation that PWH tended to prefer or lean towards managing minor symptoms at home, only seeking medical attention when severe symptoms become uncontrollable. Additionally, clinical nursing staff emphasized the importance of PWH being aware of and reflecting on their symptom management capabilities. However, they noted that differences in internal and external factors, such as individual characteristics, educational background, economic status, and family support, led to significant variations in awareness and understanding of symptom management. In the complex interaction of multiple factors, each patient ultimately adopts different coping behaviors, resulting in varying responses.

#### 3.2.1 Sub-theme 2-1: perception of self-worth

Whether being a patient or a clinical caregiver, both express the importance of understanding and appreciating individual characteristics, personal judgment, and behaviors in symptom coping and management.

##### 3.2.1.1 Sub-theme 2-1-1: self-awareness and self-regulation

The respondents’ statements demonstrate the importance of individuals coping with their symptoms. They exert their utmost effort to combat symptoms and frequently reflect on and adjust themselves to adapt to various symptoms that may occur at any time. Furthermore, from the respondents’ descriptions, we can infer that individual personality traits and relevant cognitions influence their coping and self-regulation processes regarding symptoms. In terms of personality traits, individuals who are negative, pessimistic, or even introverted often tend to adopt self-regulation methods such as avoidance, isolation, and living in despair. On the other hand, PWH with outgoing personalities, a desire for a better life, and an optimistic outlook often demonstrate a more proactive approach to addressing accompanying symptoms. They exhibit beliefs and hopes in self-management and controlling their future. At the same time, respondents not only focus on their health but also express a strong desire to protect others from infection and an awareness of cherishing every life, not just their own, but also those around them with whom they come into contact.

For example, *“There was a piece of ringworm on my neck for a period of time and then I tried a variety of medicines. But no matter what kind of medicine, it could not work. Finally, I went to a clinic, and a doctor working in this clinic said this was a piece of ringworm on my body, and gave me a kind of ointment. It was a surprise to me that medicine worked. After that, I also felt it was a signal of danger. For my skin, I was confident due to the excellent genes my parents gave me. No one in my family had skin diseases. Why was my skin so bad? For the past many years, I had never had any sores, even pimples. And I was kind of thinking that maybe there was a little bit of a problem, and I thought maybe CD4 was low at the time.”* P01.

Among all factors, nurses unanimously believe that economic status is the core constraint in all aspects. The educational background and personal personality traits of patients also play a pivotal role in their choices, a point that is highly consistent with the respondents’ statements.

For example, “*Economic status is paramount. Generally, if patients have good financial means, they are more likely to try various methods. For example, individuals with good economic conditions can opt for more advanced antiviral medications, which are more convenient to take and have fewer side effects. However, for patients with lower economic status, their choices are severely limited.*” N03.

##### 3.2.1.2 Sub-theme 2-1-2: self-coping with symptoms

Respondents employ various strategies when facing symptom occurrence, ranging from passive compromise to active resistance. Among the passive defensive strategy, there is a diverse range, including self-isolation and avoidance, seeking comfort and numbing oneself through excessive smoking, playing video games, and mahjong, or even stemming from self-abandonment or blindly discontinuing medication under the misconception of being in good health. Conversely, some respondents express a proactive attitude, refusing to be helpless or ignore the situation. They primarily demonstrate three forms of proactive and courageous coping strategies when dealing with symptoms, including self-regulation and emotional management, actively learning to acquire self-management skills, and seeking assistance from medical professionals. For instance, respondents engage in behaviors aimed at diverting attention, such as listening to music or taking naps, to alleviate stress and discomfort triggered by symptoms, and to relax themselves through psychological techniques. Additionally, respondents educate themselves or seek advice from others on coping strategies, such as self-studying relevant professional resources online or consulting medical literature to address issues. Moreover, respondents also consult experienced or well-connected fellow patients to gain insights. Of course, if symptoms become difficult to handle, they still seek help from healthcare professionals at medical institutions.

For example, “*Getting enough sleep is good for alleviating discomfort and fatigue. If I am really tired, I would take a break on-site. For example, I might take a nap on a bus or in a taxi. I need to find something to distract myself, and I cannot think about it all the time. At that time, I remembered that I might just listen to the music constantly, and then inject my own emotions into the scene described by the music.*” P05.

“*There was a paper published in the Lancet, and that is amazing, right? I think it is the most professional paper that can be published in The Lancet, so it deserves to be trusted. Moreover, patients should not spend time pondering over meaningless things, for example, when symptoms occur, you can handle them by yourself in accordance with the past way, or you need to see a doctor. All above is my attitude, you should not believe the grapevine, because it means nothing to you.*” P10.

#### 3.2.2 Sub-theme 2-2: perception of the value of social support systems

##### 3.2.2.1 Sub-theme 2-2-1: growth under the care of the state and society

In this study, respondents also mentioned their participation in various significant social activities or events at all levels over a long period of illness, under the care and support of the nation and society. This participation played a vital role in laying the foundation for their subsequent coping with diseases or symptoms. They not only acquired relevant knowledge and skills but also made more friends and established social networks. This type of social support has been immensely beneficial to them, providing comfort amidst their illnesses. However, some respondents also expressed that, compared to education activities primarily led by non-professionals, such as peer education, they trust the authority and expertise of healthcare professionals more and look forward to receiving more support and assistance from them in the future.

For example, “*I think a team that can organize some activities or lectures regularly, including sharing with each other about personal experiences, distress, and how to deal with symptoms, is very useful. Patients can learn a great deal from one another. I also took part in such activities, even though I worked as a volunteer, I think these groups are quite meaningful.”* P04.

*I have ever joined in some volunteer WeChat groups, and also worked as a volunteer. Initially, I did my best to assist other patients. But, after all, not all volunteers were professional medical staff. For example, volunteers discussed a problem together, then chatted about other unrelated matters along the way. Finally, the root cause of the problem had not been solved at all in the end. Currently, I have not spoken in the WeChat group, unless someone initiated a conversation with me, and then I shared my opinions. I think our source of information should be from healthcare providers.”* P10.

##### 3.2.2.2 Sub-theme 2-2-2: Hope evoked by medical care and nursing

With the widespread availability of antiretroviral therapy in China, along with advancements in drug research and medical technology, convenient medication options, and the gradual reduction of drug side effects, these factors have brought hope to PWH in their fight against the disease and associated symptoms. This glimmer of hope, akin to a light in the darkness, guides them to continue moving forward amidst their struggles, sustaining their belief in life.

For example*, I am still looking forward to the development of science and technology, which can restore my health. At present, there are some better drugs explored, which bring fewer side effects than the previous ones.” P06.*

*“When I was diagnosed, I was devastated when I heard about it. More than a decade has passed, and we are still well. Medical technology has also been developed, and the medical staff are very responsible for us, which makes me look forward to the future life.” P07.*

During interviews with nurses, it was common to hear them discuss the significance and value of nursing care in treating PWH. Within medical institutions, nurses are the professionals who have the most frequent contact with patients, as famously stated by Dr. Trudeau, “Sometimes to cure, often to relieve, always to comfort.” Nurses are faithful companions throughout the journey of human life, from birth to old age. They consistently provide psychological support, emotional comfort, and health guidance to patients. The spiritual strength, knowledge, and skills imparted by nurses can play a crucial role in patients’ fight against illness and symptom management, as well as in helping patients achieve good self-care at home.

For example, “*When we conduct rounds in the wards, we always ask patients how they are feeling and if they have any concerns for the day. They are very willing to discuss their worries or anxieties with us. We provide them with targeted guidance and offer some psychological comfort, and we often see improvements in their physical and mental condition*.” N01.

“*Apart from medical diagnosis and treatment, all other care tasks are primarily handled by us. We frequently provide patients with health education and guidance, and we can see the benefits that patients derive from it.*” N02.

##### 3.2.2.3 Sub-theme 2-2-3: the contradictory nature of resource supply and demand

People with HIV desire convenient access to medical services, as they may experience discomfort at any time during their prolonged illness, making this need seem more urgent. Contradictions in information or professional treatment from medical institutions are among the barriers hindering patients from building confidence in self-managing symptoms. For example, when they experience specific symptoms, they hope to contact a doctor or specialist promptly and receive remote medical services to obtain optimal guidance and treatment; however, there remains a significant gap between this ideal and reality. Even if severe symptoms arise requiring a visit to the hospital, they can feel confused and helpless. Concerns about privacy exposure in the stigmatized culture they reside in, coupled with the suboptimal choices of nearby medical resources, serve as significant factors limiting respondents’ proactive coping with symptoms.

For example, “*Although I could choose to go to the hospital when I felt uncomfortable, it was a little troublesome and not very convenient. For example, if I had a slight symptom that puzzled me, a telemedicine service would be a better option than traveling to a distant location to see a doctor. I would leave a message for the doctor and discuss something with them. I think it’s convenient, and I also would not have to run so far.*” P06.

#### 3.2.3 Sub-theme 2-3: perception of the value of interpersonal interaction systems

##### 3.2.3.1 Sub-theme 2-3-1: the motivation of love and being loved

Love and being loved are basic human needs, and they form the core content of Maslow’s theory. People living within social groups are not isolated entities. Our perceptual systems and psychological activities become enriched and fleshed out through mutual interaction. In this study, all respondents expressed the significance of love and being loved in their lives. The love from family and friends encouraged them to emerge from despair, find tranquility, and move away from fear. It is also the presence of love that makes them reluctant to abandon their existing responsibilities and obligations. Love, both given and received, becomes the most potent force supporting them as they move forward.

For example, *“Without the support of my relatives and friends, I feel like I would have collapsed. And it touched me. I mean, it touched me. During the most difficult time, my family should be my biggest support, right? I can overcome any difficulties or barriers from the disease and gain great confidence in handling symptoms, owing to their support.”* P01.

*“I feel guilty because I did not show filial piety to my parents and raise my children; they need me very much. So I think I must stick to living as long as I can insist on. Since I was stricken by the disease, even became blind, my husband have acted as my eyes and took good care of me for many years.”* P06.

##### 3.2.3.2 Sub-theme 2-3-2: satisfaction after reflection on conflicting events

During this interview, it was discovered that many respondents did not experience a fatal blow to themselves due to traumatic events; instead, they found new meaning from the adverse events they experienced or witnessed. In their view, compared to those who are experiencing extreme suffering and hovering on the brink of death, despite also enduring the invasion and torment of symptoms, they still feel satisfied, grateful, and appreciative when they receive ART and can return to everyday life. This sense of satisfaction prompts them to cherish life more, actively cooperate with treatment, and engage in self-management.

For example, *“Looking! The condition of that patient in the ward next to mine is more serious than mine. I think I am lucky and have a good attitude. I will actively coordinate with the treatment and deal with any discomfort as soon as possible.”* P01.

*“Anyway, my uncle was also a doctor, but died at 37. And my brother died at the age of 57. I am 58 years old, have lived many years longer than them, and I am satisfied.”* P07.

##### 3.2.3.3 Sub-theme 2-3-3: exemplification of successful role models

Some participants in this study reported that they guide themselves in actively coping with their illness and symptoms by following the developmental trajectories or stories of successful individuals. This highlights the significant role of successful role models in providing psychological and emotional support to patients.

For example, *“I seek out individuals in life who are particularly positive or have shown remarkable growth through their struggles. I draw inspiration from the development trajectories of these role models to motivate myself to move forward and persevere.”* P05.

##### 3.2.3.4 Sub-theme 2-3-4: the burden within the culture of illness

Due to the impact of societal discrimination, culture, and stereotypical impressions of this disease, PWH often develop a unique sense of HIV-related stigma, which can further exacerbate their physical and mental health and have adverse effects on their coping attitudes, beliefs, and health behaviors. The participants in this study openly admitted that being immersed in the culture of HIV, whether in their work, daily life, or medical encounters, this burden follows them everywhere. They constantly feel on edge, fearing exposure of their illness and subsequent social rejection and discrimination from those around them. Particularly when symptoms occur, silently enduring or privately managing them, or seeking medical advice discreetly, are all real-life portrayals of PWH under the influence of disease culture.

For example, “*I needed to see a doctor when I felt uncomfortable and could not handle it by myself, but I dared not go to another hospital, because I could not tell others that I had AIDS.*” P02.

“*After the diagnosis, I went back to work very soon until my leader talked to me. They persuaded me to retreat and did not allow me to go to work, later I could only stay at home all the time.*” P04.

“*I discarded any materials involving illness-related information before coming back to my house, in order to keep my roommates from finding out something.*” P10.

## 4 Discussion

The symptoms of PWH are manifested in two aspects: physical and psychological. Typical physical and physiological reactions include fever, fatigue, nutritional problems, and side effects of ART, such as dizziness, nausea, vomiting, headache, diarrhea, muscle pain or joint pain, and fatigue in accordance with previous studies, as well as emotional or psychological reactions include pain, anxiety, fear, and depression, and a change in role function (Zhu et al., 2023; Zhu et al., 2019). For healthcare providers, understanding the cause of their symptoms and evaluating their personal values from the patient’s perspective is significant. The complexity, interaction, and dynamics of the symptoms arise not only from multiple sources but are also intricately linked to the patient’s ecosystem, which encompasses the natural environment, social factors, cultural influences, and other relevant elements. PWH’s perception and interpretation of symptoms vary widely due to the same symptoms derived from different sources or different individuals with similar symptoms. Additionally, psychological problems such as exhaustion, spiritlessness, and stress are closely related to their physical symptoms. It’s equally necessary that patients learn to live with HIV symptoms, comorbidities, and side effects. In their ongoing struggle for survival, individuals endure pain that may be incomprehensible to others. However, their optimistic attitudes, philosophical outlooks that embrace fate, and respect for their self-worth are seamlessly integrated into their real-world behavioral practices. If the confusion and uncertainties experienced by them can be clearly addressed in future clinical interventions and health education systems, it would significantly enhance their understanding of the disease. This, in turn, would inspire proactive health behaviors and improve their overall quality of life.

Although the SSMM-HIV model introduces the concept of PSM into the symptom management pathway for PWH, the understanding of PSM has not yet been thoroughly explored so far (Spirig et al., 2005). This highlights the need to acknowledge that there are still underlying mechanisms that warrant further discussion and research in the practice of symptom management and the enhancement of quality of life for PWH. We need to understand how to leverage these internal mechanisms to achieve the ultimate goal of symptom management and improve clinical outcomes (Fierz et al., 2013). Researchers found that PWH pay attention to symptoms beyond what is typically understood as “relief” (Liu et al., 2018), which involves using clinical parameters to assess the success of symptom handling or management by healthcare providers. However, present clinical parameters may not fully capture the patient’s perspective. It is only by integrating symptom management into daily life that we can truly understand and address it in a meaningful way. The perceived manageability of symptoms is particularly significant for PWH in shaping their life’s meaning. This study demonstrated that PWH exhibited strong personal awareness and introspective abilities in managing symptoms and coping processes. PWH with different characteristics may have other personal roles and value orientations. For example, respondents with higher levels of education seem to possess stronger intrinsic initiative and are more capable of adopting various coping measures when facing symptoms. However, for PWH with lower levels of education, they are more inclined to believe that all they can do is persevere when symptoms strike, questioning whether there are optimization strategies that could improve the situation; they may feel inadequate or powerless. In the interview section targeting nurses, respondents also expressed similar views. Previous evidence confirms that PWH with limited education in China demonstrate significantly constrained symptom management agency, often defaulting to passive endurance during symptom exacerbation due to gaps in self-efficacy and health literacy (Ebersole et al., 2021). Crucially, these individuals express empirical uncertainty regarding the existence or accessibility of optimization strategies, manifesting as perceived helplessness (Yang et al., 2024). This aligns with frontline clinical observations: nurses consistently report that patients with lower educational attainment disproportionately exhibit therapeutic passivity and hesitancy in articulating symptom management needs (Tarantino et al., 2024). Additionally, Haase and his team revealed that patients’ behavioral strategies in coping with symptoms could be divided into two major types: courage strategies (protective) and defensive strategies (dangerous) (Haase et al., 2014), which is influenced by individual cognitive levels (Leventhal et al., 1998). Our study presented that personal characteristics were also key factors affecting coping behaviors. Compared with those isolated and pessimistic PWH, those who are optimistic, sociable, and open-minded are more likely to accept the reality of the disease and play a positive personal role in the fight against the disease. This implies that healthcare providers and researchers should not be helpless facing intrinsic uncontrollable factors of PWH, but incorporate the assessment of an individual’s characteristics into the future symptoms management practice, to develop a personalized management path, medical support, and guidance based on a comprehensive evaluation of the individual, which will be a significant value for clinical practice.

Moreover, the profound and extensive history and culture of China have nurtured the essence of Chinese philosophy, which profoundly influences modern people’s concepts of survival, medical treatment, and death. The thoughts of Confucianism, Taoism, Legalism, and various other schools have far-reaching impacts. Traditional Chinese philosophical concepts-notably “the unity of heaven and humanity,” “primacy of human life,” and “harmony with natural order” -are deeply embedded in China’s cultural ethos (Dwyer et al., 2024). For example, PWH’s avoidance of aggressive symptom control (e.g., rejecting analgesic escalation) embodied Taoist ‘non-contention with natural forces’. By framing symptoms as ‘seasonal winds that must run their course, they reduced cognitive dissonance through cosmic fatalism. These principles historically bridged medicine and philosophy in Chinese civilization, shaping distinctive approaches to health management. Consequently, PWH in China often exhibit restraint, non-confrontation, adaptive acceptance, and resilience when navigating disease and suffering, which aligns with Chen’s opinion that Chinese philosophies and religions strongly influence the Chinese way of living and thinking about health and healthcare. Therefore, healthcare providers must integrate cultural information with clinical assessment to provide culturally sensitive care for patients’ symptoms. In coping with symptoms, the respondents in this study maintained an attitude of doing what they could within their means and of conforming to the laws of nature. Most of them have experienced a gradual acceptance of their illness and have learned to live in harmony with it through various self-regulation methods. In this process, they have deepened their self-affirmation and realized that the path to survival is akin to self-cultivation. Additionally, in their view, the support of national and societal policies, along with the continuous advancement of medical technology, is a blessing. They believe that only when self and external assistance are harmoniously unified can they achieve ultimate survival and happiness. The respondents look forward to the emergence of better treatment drugs or technologies in the future and hope for more policies that support their medical journey, helping them find hope and overcome the challenges they face.

Whether it be filial piety or the foundation of benevolence, it has become the source of interpersonal relationships, family values, and affection among Chinese people. The respondents in this study elaborated to varying degrees on the love, care, bonds, and guilt stemming from familial or friendly relationships. Complex emotions and sentiments envelop their lives, yet they serve as the driving force for their struggles and the reason for their continued existence. The Confucian saying “the way of heaven, earth, and humanity’s three powers,” which describes the relationship of “born by heaven, nourished by earth, developed by humans,” embodies the concept of the perfect unity of heaven and humanity, the body and mind of individuals, interpersonal relationships, and the harmony between humans and nature (Zhu, 2014). Under the influence of these thoughts, the respondents have developed their attitudes and beliefs regarding symptom management. They perceive the concept of PSM as centered on the individual, supported by a robust foundation of state and societal support, and reliant on a compassionate interpersonal support system to better cope with distressing symptoms. They believe that optimal results in maintaining self-health and effectively managing symptoms can only be achieved through the harmonious interaction of these three elements. Furthermore, the findings of this study will promote the incorporation of cultural attributes and social factors in China as crucial considerations in the formulation and implementation of systems and initiatives. Therefore, it is both promising and urgent to develop scientific, professional, and practical medical assistance programs for PWH in the future. Researchers and clinical experts in China have been advancing on this challenging path, utilizing modern information technology and scientific evidence to actively explore home care, symptom management, and medication guidance for PWH (Han et al., 2021). Additionally, how to change the discriminatory environment for PWH by using the unique Chinese cultural strategies and reduce their inner burden from this disease will be a hot topic in the future. Redirecting Taoist non-contention from fatalism to mindful self-regulation and Family-Embedded Symptom Stewardship, rooted in leveraging filial piety dynamics to transform passive endurance into collective action, may be a choice.

When contextualized globally, our findings reveal both convergence and divergence with symptom management literature. The centrality of family-based resource mobilization contrasts with community-centric approaches observed in sub-Saharan Africa (Mankhokwe et al., 2023)yet mirrors Vietnamese patterns of stigma avoidance (Alemu et al., 2021). Similarly, while symptom interpretation as moral suffering parallels Kenyan narratives, the absence of spiritual framing in Chinese accounts highlights the role of secular collectivist values. Most notably, we identify ‘silent self-efficacy’ as a culturally salient manageability strategy—distinct from Brazil’s advocacy-based approaches (Iribarren et al., 2018) or Uganda’s collective coping (Larsson et al., 2023). This suggests that SSMM-HIV’s mediator pathways require cultural adaptation when applied to high-stigma settings, particularly regarding how structural discrimination* suppresses public resource utilization.”

Our findings also demonstrate significant theoretical alignment with the SSMM-HIV model while revealing critical cultural extensions relevant to the Chinese context. For example, the theme of self-regulation aligns with the SSMM-HIV’s emphasis on personal agency in symptom management. Participants’ experiences of confusion about symptom origins and anxiety regarding symptom progression reflect the cognitive and emotional challenges in symptom management that the SSMM-HIV model seeks to address. The perception of social support systems, including growth under the care of the state and society, as well as the hope evoked by medical care and nursing, aligns with the SSMM-HIV model’s focus on social support and resource utilization in symptom management. Additionally, the contradictory nature of resource supply and demand highlights the ongoing tension between available resources and patient needs, which is an important consideration within the SSMM-HIV framework. Overall, these findings provide valuable insights into the symptom experiences of the study population and offer a practical perspective on the application of the SSMM-HIV model in real-world settings.

## 5 Researcher positionality statement and introspection

Our team’s extensive clinical engagement in HIV care inherently shaped preconceptions about symptom trajectories in PLWH. To leverage this expertise while mitigating interpretive bias, we adopted interpretative phenomenological analysis (IPA)—a methodology expressly designed to explore meaning-making in lived experiences while acknowledging researcher subjectivity as integral to co-constructed understanding. Firstly, structured bracketing involved documenting presuppositions pre-interview and suspending them during data collection using open-ended probes; secondly, iterative reflexivity through biweekly debriefings, challenging interpretations against disconfirming evidence, and independent coding by non-clinical methodologists; and triangulated trustworthiness via member validation, peer examination of decision trails, and thick contextualization within China’s syndemic of HIV stigma and familial collectivism. Crucially, our clinical backgrounds enabled semantic precision during co-interpretation; participants confirmed that researcher familiarity with medical idioms helped articulate previously ineffable experiences. This exemplifies IPA’s epistemological stance, where meaning emerges through dialogical engagement that fuses researcher-participant horizons, yielding frameworks that reflect neither pure clinician logic nor unmediated patient voice, but authentically negotiated understandings of symptom manageability.

## 6 Limitations

This study identified pertinent issues that warrant further investigation in large-scale, multimethod studies, despite using a small, selected sample. The information we collected helped us understand perceptions of symptoms and self-management among PWH in China from two groups: PWH and nurses. Although all participants were recruited from the capital city of China, their views might not be representative of individuals in other parts of the country. They may not reflect the experiences of PWH in rural or ethnically diverse regions of China. Furthermore, due to the limited number of young female patients, the information obtained in this study may not be sufficient and still requires further enrichment and improvement. Third, this study was conducted in a clinic or hospital setting, which may have influenced the volunteers’ freedom to respond to the research questions, as clinical procedures were conducted there. We fully agree that subthemes with limited participant concordance require explicit qualification to avoid overgeneralization. However, the subtheme ‘satisfaction after reflecting on conflicting events’ emerged in 7 of 11 participants (63.6%) who described treatment adversity. While not universal, its presence across diverse demographic strata suggests contextual significance for those reconciling therapeutic setbacks with cultural values of endurance.

Our clinical backgrounds may have privileged medically legible symptoms over embodied distress. However, this bias was productively leveraged during member checking when participants validated that clinician-understood terminology improved communication of their experiences. The tension between emic and etic perspectives ultimately generated richer theoretical insights into SSMM-HIV’s clinical applicability.”

## 7 Conclusion

The symptoms of PWH exhibit complexity, interactivity, dynamics, and multifaceted origins, leading to differences in perception and interpretation among patients. This results in cognitive confusion and chaos within this population, providing important insights for future exploration in clinical health education. Simultaneously, the characterization and coping strategies for PWH’s symptoms are intertwined with their personal characteristics, life experiences, cultural backgrounds, etc., leading to varying interpretations and expressions of beliefs and attitudes towards symptom management. This study provides a systematic exposition and conceptual delineation of PSM, establishing a rigorous scientific framework for investigating its intrinsic motivational mechanisms. Furthermore, it enriches the empirical basis for future clinical research aimed at enhancing symptom management and quality of life among PWH. By elucidating culture-specific coping strategies and their implications for HIV care, this research significantly advances the understanding of PSM within the Chinese context. China’s millennia-old culture profoundly influenced PWH and has developed unique perspectives on survival, medical treatment, illness stigma, and death. Exploring intervention strategies grounded in Chinese philosophical thought and achieving home-based self-management practices with medical professionalism at the core are their greatest hope. While this study elucidates the cultural underpinnings of symptom manageability among Chinese PWH, longitudinal mixed-methods studies should track how PSM evolves across the HIV continuum further.

## Data Availability

The data analyzed in this study is subject to the following licenses/restrictions: Because the subjects of our investigation belong to vulnerable populations, we deliberately disclosed in the informed consent that we cannot reveal their information. Requests to access these datasets should be directed to lianlian12600@126.com.
